# Sex and racial differences in cardiovascular disease risk in patients with atrial fibrillation

**DOI:** 10.1371/journal.pone.0222147

**Published:** 2019-09-04

**Authors:** Wesley T. O’Neal, Aniqa B. Alam, Pratik B. Sandesara, J’Neka S. Claxton, Richard F. MacLehose, Lin Y. Chen, Lindsay G. S. Bengtson, Alanna M. Chamberlain, Faye L. Norby, Pamela L. Lutsey, Alvaro Alonso

**Affiliations:** 1 Department of Medicine, Division of Cardiology, Emory University School of Medicine, Atlanta, GA, United States of America; 2 Department of Epidemiology, Rollins School of Public Health, Emory University, Atlanta, GA, United States of America; 3 Division of Epidemiology and Community Health, School of Public Health, University of Minnesota, Minneapolis, Minnesota, United States of America; 4 Cardiovascular Division, Department of Medicine, University of Minnesota Medical School, Minneapolis, Minnesota, United States of America; 5 Health Economics and Outcomes Research, Life Sciences, Optum, Eden Prairie, Minnesota, United States of America; 6 Department of Health Sciences Research, Mayo Clinic, Rochester, Minnesota, United States of America; Medizinische Universitat Innsbruck, AUSTRIA

## Abstract

**Background:**

Outcomes among atrial fibrillation (AF) patients may differ according to race/ethnicity and sex due to differences in biology, the prevalence of cardiovascular risk factors, and the use and effectiveness of AF treatments. We aimed to characterize patterns of cardiovascular risk across subgroups of AF patients by sex and race/ethnicity, since doing so may provide opportunities to identify interventions. We also evaluated whether these patterns changed over time.

**Methods:**

We utilized administrative claims data from the Optum Clinformatics® Datamart database from 2009 to 2015. Patients with AF with ≥6 months of enrollment prior to the first non-valvular AF diagnosis were included in the analysis. Final analysis utilized Cox proportional hazard models to estimate adjusted hazard ratios (HR) and 95% confidence intervals (CI) for cardiovascular outcomes stratified by sex and race/ethnicity. An additional analysis stratified outcomes by calendar year of AF diagnosis to evaluate changes in outcomes over time.

**Results:**

In a cohort of 380,636 AF patients, women had a higher risk of ischemic stroke [HR (95% CI): 1.25 (1.19, 1.31)] and lower risk of heart failure and myocardial infarction [HR (95% CI): 0.91 (0.88, 0.94) and 0.81 (0.77, 0.86), respectively)] compared to men. Black patients had elevated risk across all endpoints compared to whites, while Hispanics and Asian Americans showed no significant differences in any outcome compared to white patients. These sex and race/ethnic differences did not change over time.

**Conclusions:**

We found sex and race/ethnic differences in risk of cardiovascular outcomes among AF patients, without evidence of improvement over time.

## Introduction

Women and black patients with atrial fibrillation (AF) have been reported to have higher rates of stroke and other cardiovascular diseases compared with their male and white counterparts.[1, 2] However, prior studies have not examined these differences across cardiovascular outcomes in sufficiently large populations. Moreover, whether these race/ethnic and sex differences have decreased or increased over time is unknown. Characterizing patterns of risk across subgroups of AF patients and their change over time can identify opportunities for intervention, leading to amelioration of existing sex and race/ethnic differences.

The purpose of this analysis was to examine whether rates of cardiovascular outcomes among patients with AF varied by sex and race/ethnicity, and whether these rates changed over time using data from a large administrative claims database.

## Methods

### Study population

This study used administrative claims data from the Optum Clinformatics® Datamart database between January 1, 2009 and September 30, 2015.[3] The database is comprised of de-identified, patient-level data regarding plan enrollment and medical and pharmacy claims from health care providers. This analysis included health plan enrollees with ≥6 months of enrollment prior to the first non-valvular AF diagnosis. AF was defined by International Classification of Diseases Ninth Revision, Clinical Modification (ICD-9-CM) codes 427.31 or 427.32 in any position on an inpatient claim or on two consecutive outpatient claims at least 7 days but less than 1 year apart, and without any inpatient diagnosis of mitral stenosis (ICD-9-CM 394.0) or mitral valve disorder (ICD-9-CM 424.0). The AF diagnosis date was defined as the earliest of the discharge date of the inpatient claim or the service date of the second outpatient claim.[4] The Institutional Review Board of Emory University (Atlanta, GA, USA) approved the present study, which provided a waiver of informed consent for the analysis of these deidentified data.

### Covariate assessment

Age at the time of AF diagnosis, sex (female/male), education level, and race/ethnicity were recorded at the time of health plan enrollment. Age at time of AF diagnosis was modeled continuously. Age was also categorized into the following groups for age-stratified analysis: age ≤70, 70 < age ≤ 80, and age > 80. Education was categorized into less than 12^th^ grade, high school diploma, less than Bachelor Degree, Bachelor Degree and beyond, and unknown. Race/ethnicity was categorized as white, black, Hispanic, and Asian American and collected from public records in approximately 30% of participants or imputed using commercial software (E-Tech by Ethnic Technologies) in the rest. This method of imputation has been previously validated and has demonstrated 48% sensitivity and 97% specificity of classifying black race/ethnicity (relative to white).[5] ICD-9-CM diagnosis codes in any position from inpatient or outpatient claims were used to detect the presence of comorbid conditions prior to AF diagnosis and compute CHA_2_DS_2_-VASc scores (congestive heart failure, hypertension, age ≥75 years, diabetes mellitus, stroke/transient ischemic attack, vascular disease, and age 65–75 years) for each patient.[6] Sex was removed from CHA_2_DS_2_-VASc score calculations to account for the presence of sex as an independent variable in the model.

### Endpoint definition

Stroke, myocardial infarction, and heart failure were defined by the presence of inpatient ICD-9-CM codes in the primary position. For patients whose AF was diagnosed from inpatient claims, follow-up began the day of discharge; therefore, stroke, heart failure, or myocardial infarction occurring during the index hospitalization were not included as endpoints. All ICD-9-CM codes used for detecting study endpoints and comorbid conditions are provided in **S1 Table**.

### Statistical analysis

Cox regression was used to estimate hazard ratios (HR) and 95% confidence intervals (CI) for each outcome by sex and race/ethnicity, adjusting for age, education, and CHA_2_DS_2_-VASc scores. Time to event was defined as days elapsed since AF diagnosis to the occurrence of the event, database disenrollment or September 30, 2015, whichever occurred earlier. The primary analysis included all eligible patients. We also conducted age-stratified analysis based on the age groups previously defined, and used interactions terms for age-sex and age-race to test for changes in associations across age. In an additional analysis, we stratified the cohort by calendar year of AF diagnosis to evaluate changes in cardiovascular outcomes over time. For this analysis, participants were followed up for one year after AF diagnosis, with censoring occurring at the earlier of 365 days or disenrollment. Year-specific Cox regression models were used to estimate adjusted hazard ratios for each outcome by sex and race/ethnicity. Each calendar year began in January and ended in December, except for the year of 2014. Because overall follow-up ends on September 30, 2015, for the 2014 calendar year cohort we only included those diagnosed with AF January 1, 2014 through September 30, 2014. Interaction terms of calendar year of AF diagnosis with sex and race/ethnicity in the analysis including the entire sample were used to test changes in associations over time. SAS Version 9.4 (Cary, NC) was used for all analyses.

## Results

A total of 380,636 participants (mean age: 73 years; 45% women; 82% white; 9% black; 7% Hispanic; 2% Asian American) were included. **Table 1** provides patient characteristics at the time of AF diagnosis by race/ethnicity and sex. Compared to men, women in this cohort were more likely to have hypertension, be over the age of 75, and have had a previous stroke. Among the racial and ethnic groups, black patients were more likely to have congestive heart failure, have hypertension, and have had a previous stroke, even though they were generally younger than their counterparts. Hispanic patients were more likely to have diabetes and vascular disease. Mean time to event in days for the study endpoints for all participants were as follows: stroke, 699; heart failure, 688; and myocardial infarction, 700. Heart failure was the most common outcome of the cardiovascular endpoints among all patients.

**Table 1:** Characteristics of patients with atrial fibrillation by sex and race/ethnicity, Optum Clinformatics 2009–2015.

	Men	Women	Whites	Blacks	Hispanics	Asian Americans
**N.**	208,256	172,380	313,042	32,095	27,453	8,046
Age at AF Diagnosis, years*	70.8 (12.3)	75.2 (11.0)	73.0 (11.8)	71.0 (12.6)	73.3 (12.4)	73.6 (12.2)
**Women, %**			44.7	50.0	46.7	42.7
**Education, %**						
**Less than 12**^**th**^ **grade**	0.6	0.7	0.2	0.4	4.9	1.0
**High school**	29.5	31.3	26.9	54.1	44.0	23.4
**Less than Bachelor** **Degree**	55.5	55.8	58.2	41.4	43.4	53.3
**Bachelor Degree and beyond**	14.1	12.1	14.4	3.9	7.4	22.2
**Unknown**	0.3	0.3	0.3	0.1	0.3	0.1
**Race/ethnicity, %**						
**Whites**	83.1	81.3				
**Blacks**	7.7	9.3				
**Hispanics**	7.0	7.4				
**Asians**	2.2	2.0				
CHA2DS2-VASc Scores Comorbidities, %*ǂ	3.4 (2.0)	3.9 (1.9)	3.6 (1.9)	3.8 (1.9)	4.0 (2.0)	3.7 (2.0)
**Congestive heart failure**	33.1	34.1	32.3	41.7	38.5	31.1
**Hypertension**	80.8	85.6	82.0	88.9	87.3	83.1
**Age ≥ 75**	44.4	60.6	52.0	45.4	55.2	55.2
**Age 65–74**	28.6	24.1	26.5	27.9	25.5	26.5
**Previous stroke**	24.7	29.2	26.2	30.0	29.3	27.7
**Vascular disease**	31.5	31.4	30.7	34.0	37.8	30.4
**Diabetes mellitus**	36.1	34.4	33.0	44.8	48.4	42.6

* Mean (Standard Deviation)

ǂ CHA_2_DS_2_-VASc without sex

Crude rates of ischemic stroke and heart failure were higher in women than men, while men had higher rates of myocardial infarction than women (**Table 2**). In multivariable Cox models, however, women had a higher rate of ischemic stroke [HR (95% CI): 1.25 (1.19, 1.31)], but a lower rate of heart failure [HR (95% CI): 0.91 (0.88, 0.94)] and myocardial infarction [HR (95% CI): 0.81 (0.77, 0.86)] compared with men. When stratified by age, only women above the age of 70 had a higher rate of stroke compared to men (**S2 Table**), with the association growing stronger as age increased (p-value for age-sex interaction for stroke: p < .0001). Age did not appear to influence rates of heart failure and myocardial infarction in women compared to men (**S3–S4 Tables**).

**Table 2:** Associations of sex and race/ethnicity with incidence of ischemic stroke, heart failure, and myocardial infarction in patients with atrial fibrillation, Optum Clinformatics 2009–2015.

	Men	Women	Whites	Blacks	Hispanics	Asian Americans
**N.**	208,256	172,380	313,042	32,095	27,453	8,046
**Ischemic Stroke**						
**Years follow-up, median (25th %tile-75th %tile)**	1.4 (0.6–2.9)	1.4 (0.5–2.9)	1.4 (0.6–2.9)	1.2 (0.5–2.6)	1.5 (0.6–3.0)	1.5 (0.6–2.9)
**Person-Years of Follow-up**	399,921	328,703	602,989	55,639	54,431	15,565
**N. events**	3,251	3984	5,671	768	625	171
Crude IR^*^	8.1	12.1	9.4	13.8	11.5	11.0
HR (95%CI)^**^	1 (ref)	1.25 (1.19, 1.31)	1 (ref)	1.44 (1.33, 1.55)	1.09 (1.00, 1.19)	1.12 (0.96, 1.30)
**Heart failure**						
**Years follow-up, median (25th %tile-75th %tile)**	1.4 (0.5–2.9)	1.4 (0.5–2.9)	1.4 (0.5–2.9)	1.2 (0.4–2.5)	1.4 (0.6–2.9)	1.5 (0.6–2.9)
**Person-Years of Follow-up**	393,421	323,939	594,354	54,170	53,442	15,375
**N. events**	9264	7994	13,439	2090	1422	307
Crude IR^*^	23.5	24.7	22.6	38.6	26.6	20.0
HR (95%CI)^**^	1 (ref)	0.91 (0.88, 0.94)	1 (ref)	1.45 (1.38, 1.52)	0.95 (0.90, 1.01)	0.84 (0.75, 0.94)
**Myocardial infarction**						
**Years follow-up, median (25th %tile-75th %tile)**	1.4 (0.6–2.9)	1.4 (0.6–2.9)	1.4 (0.6–2.9)	1.2 (0.5–2.6)	1.5 (0.6–3.0)	1.5 (0.6–2.9)
**Person-Years of Follow-up**	399,723	330,052	603,902	55,671	54,586	15,607
**N. events**	3199	2386	4490	538	450	107
Crude IR^*^	8.0	7.2	7.4	9.7	8.2	6.9
HR (95%CI)^**^	1 (ref)	0.81 (0.77, 0.86)	1 (ref)	1.12 (1.03, 1.23)	0.95 (0.86, 1.05)	0.89 (0.73, 1.08)

IR, incidence rate; HR, hazard ratio; CI, confidence interval.

*Per 1,000 person-years

**Cox model adjusted for age, sex, race/ethnicity, education and CHA_2_DS_2_-VASc scores

In the context of race/ethnicity, black patients presented with the highest HRs, particularly with ischemic stroke [HR (95% CI): 1.44 (1.33, 1.55)] and heart failure [HR (95% CI): 1.45 (1.38, 1.52)] (**Table 2**). The effect was weaker, albeit still significant, for black patients with AF and their risk for myocardial infarction [HR (95% CI): 1.12 (1.03, 1.23)]. Compared to whites, Hispanic patients had a marginally higher rate of ischemic stroke, but did not differ significantly in rates of heart failure and myocardial infarction. Differences between whites and Asian Americans were not significant, save for a protective effect among Asian Americans for heart failure. When stratified by age, younger black patients with AF had higher rates of all outcomes than their older counterparts when compared against white patients with AF (**S2–S4 Tables**). Younger Hispanic patients also appeared to follow this trend, albeit to a lesser degree. Overall, there was evidence of age-race interaction for all cardiovascular endpoints (p-values for age-race interaction ≤ 0.001 for all endpoints).

**Fig 1:**
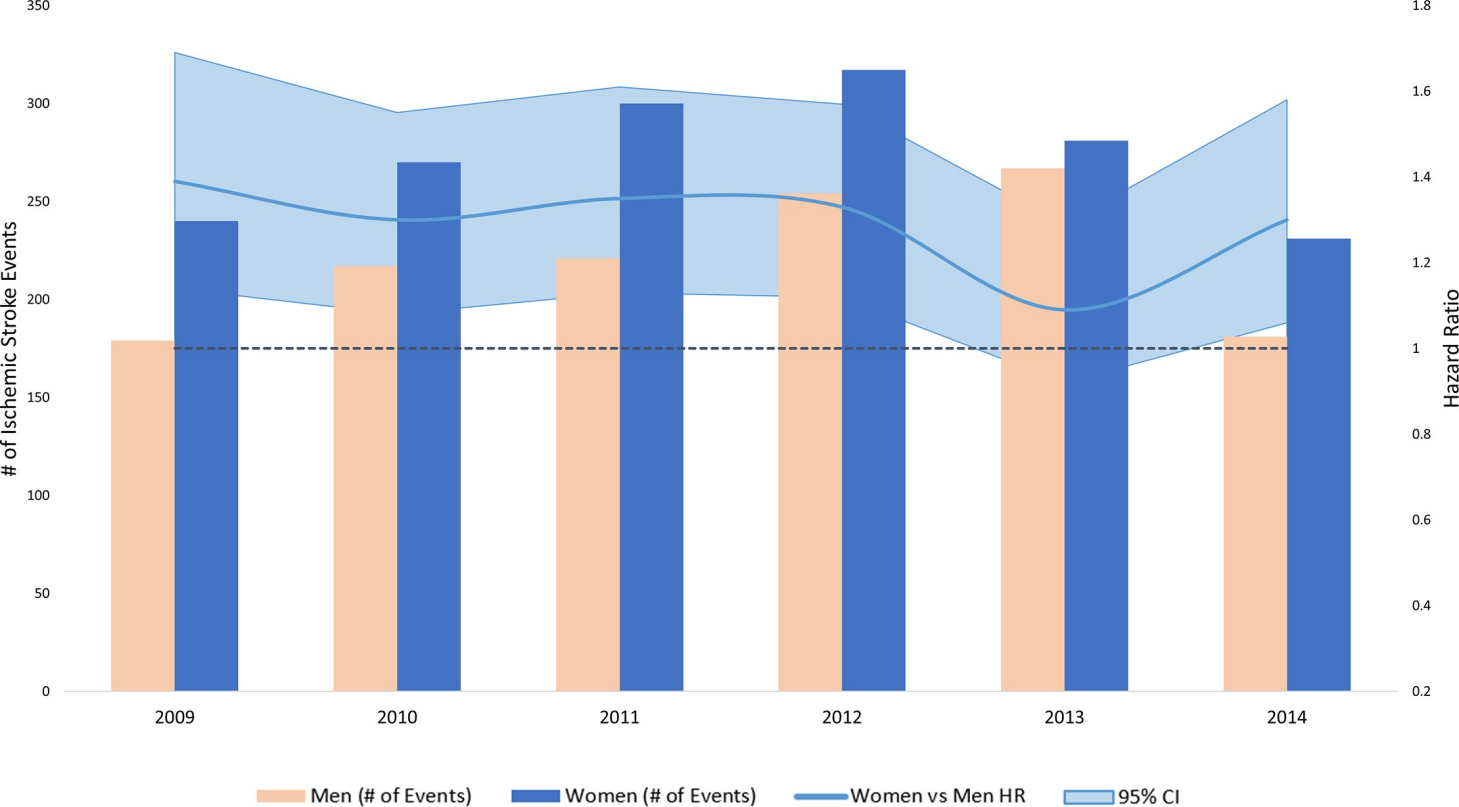
Population counts by sex and adjusted HRs (95% CI) for associations of sex with incidence of ischemic stroke in patients with atrial fibrillation by year, Optum Clinformatics 2009–2015.

**Fig 2:**
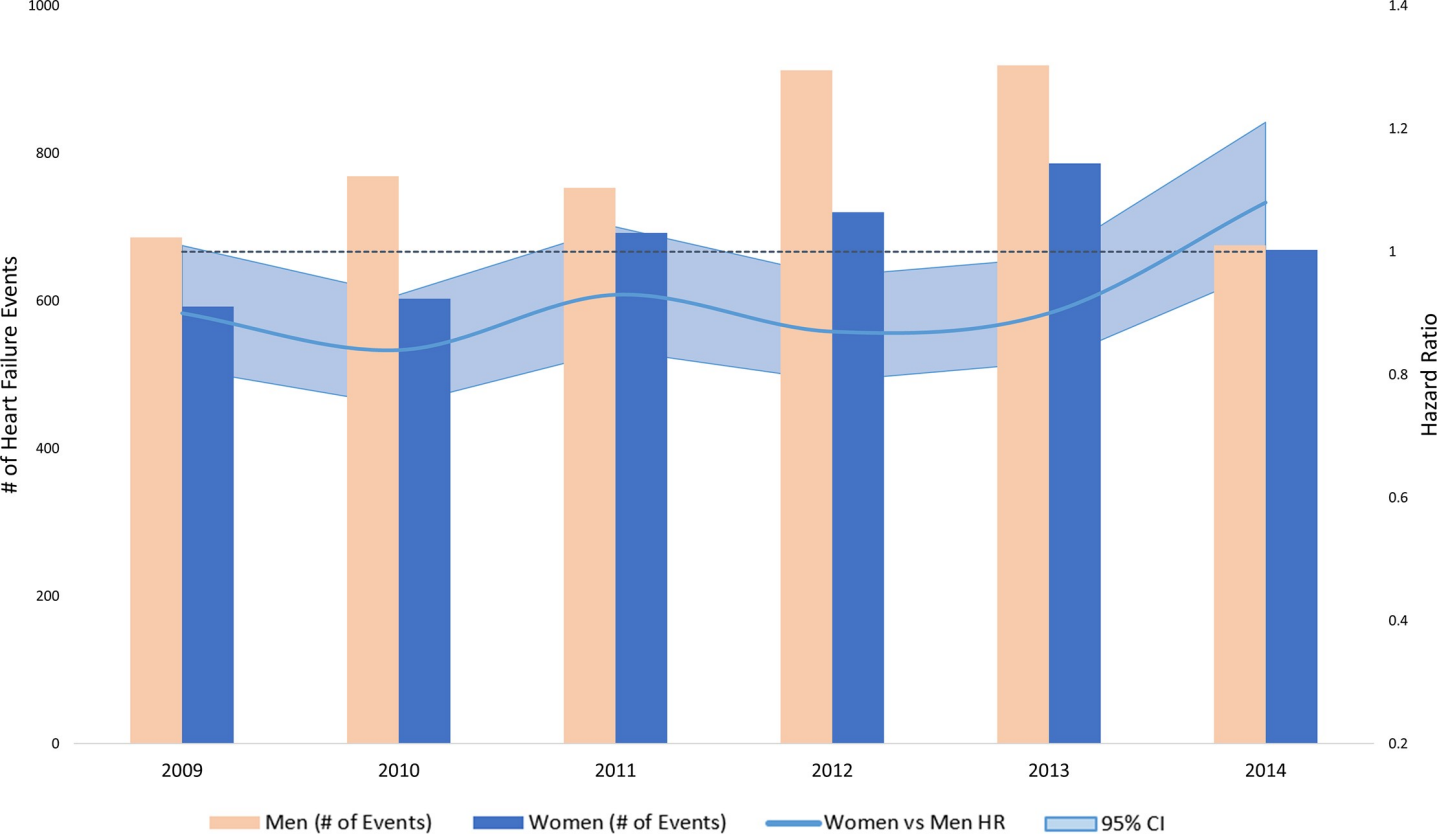
Population counts by sex and adjusted HRs (95% CI) for associations of sex with incidence of heart failure in patients with atrial fibrillation by year, Optum Clinformatics 2009–2015.

**Fig 3:**
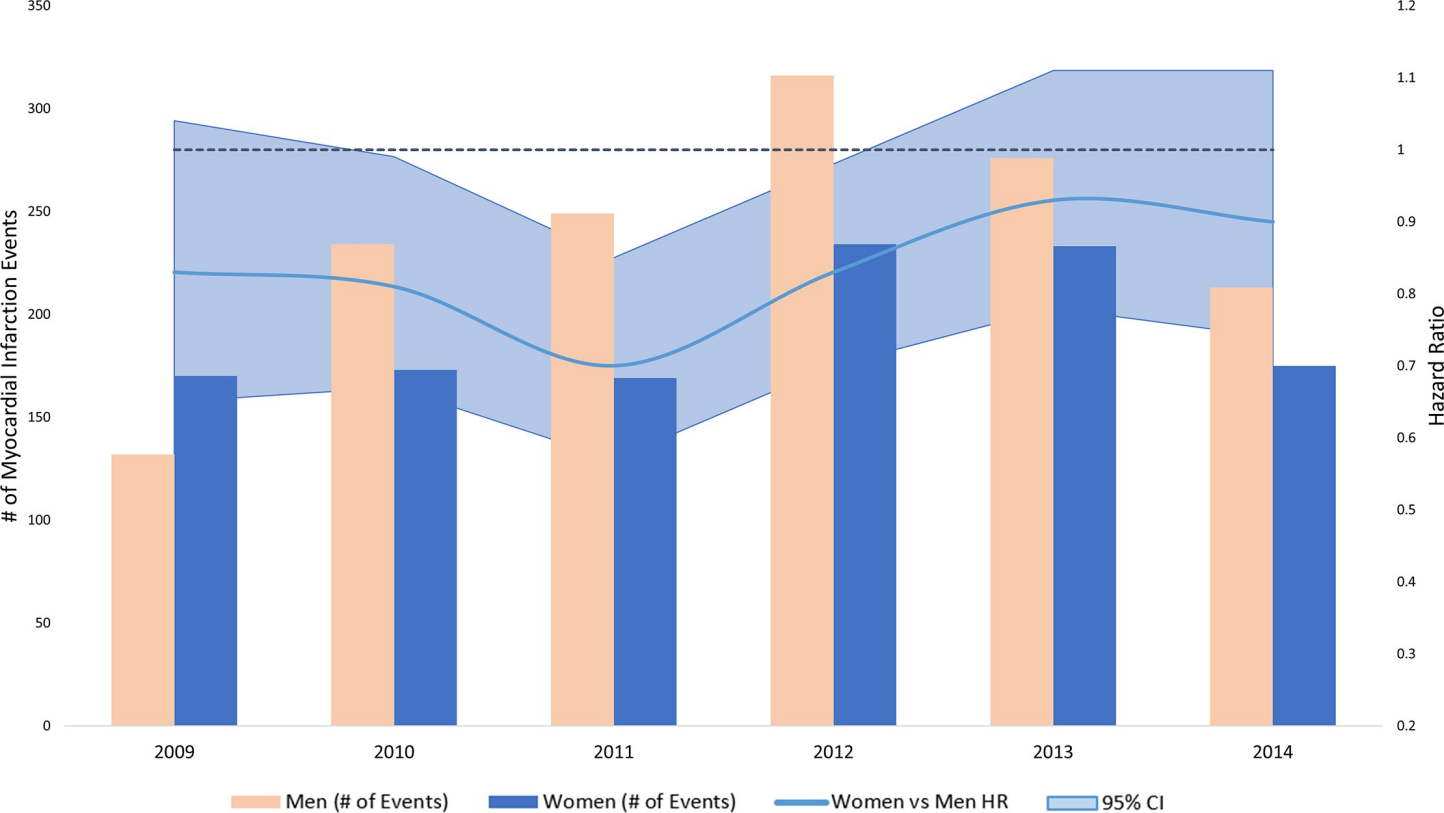
Population counts by sex and adjusted HRs (95% CI) for associations of sex with incidence of myocardial infarction in patients with atrial fibrillation by year, Optum Clinformatics 2009–2015.

**Fig 4:**
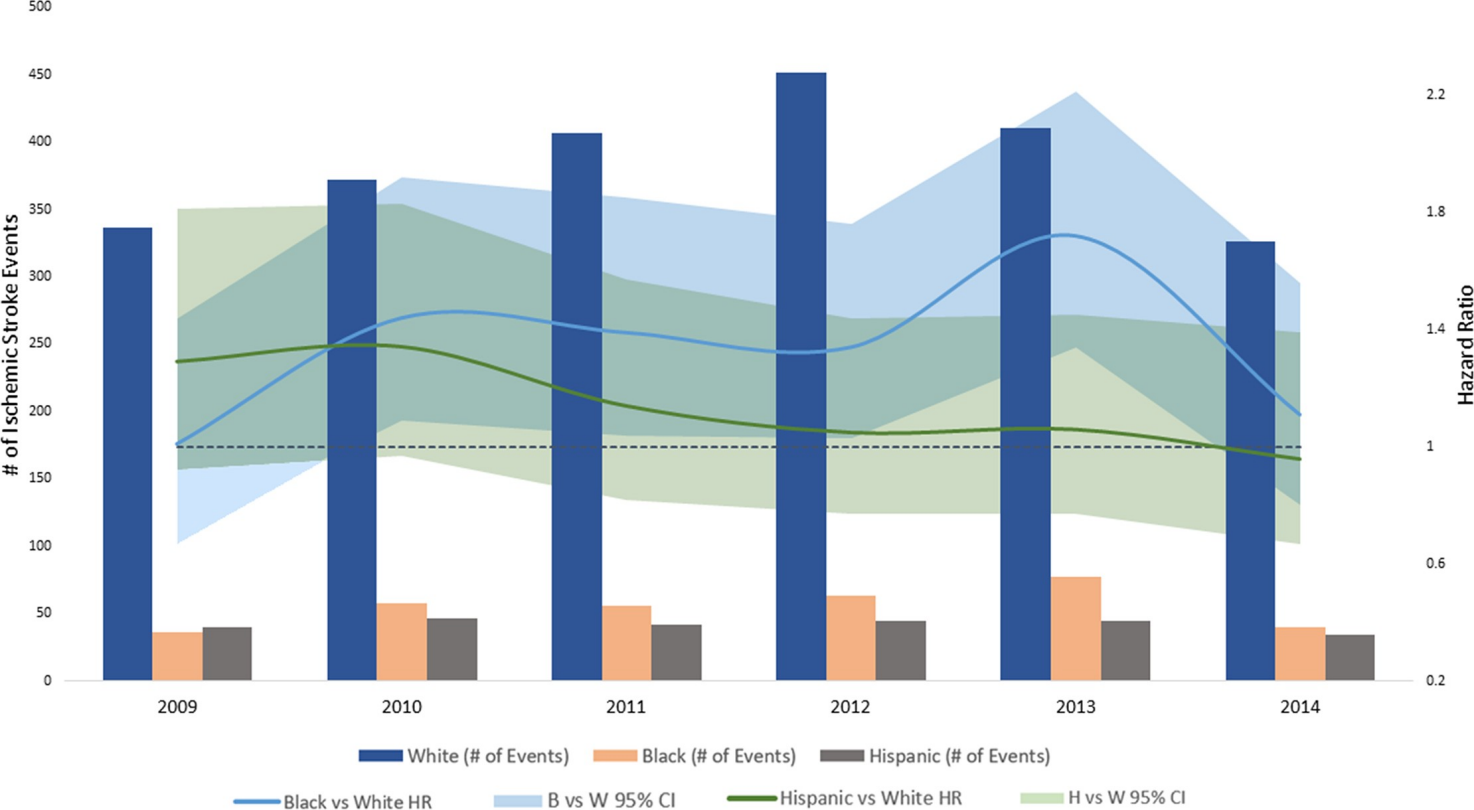
Population counts by race/ethnicity and adjusted HRs (95% CI) for associations of race/ethnicity with incidence of ischemic stroke in patients with atrial fibrillation by year, Optum Clinformatics 2009–2015.

**Fig 5:**
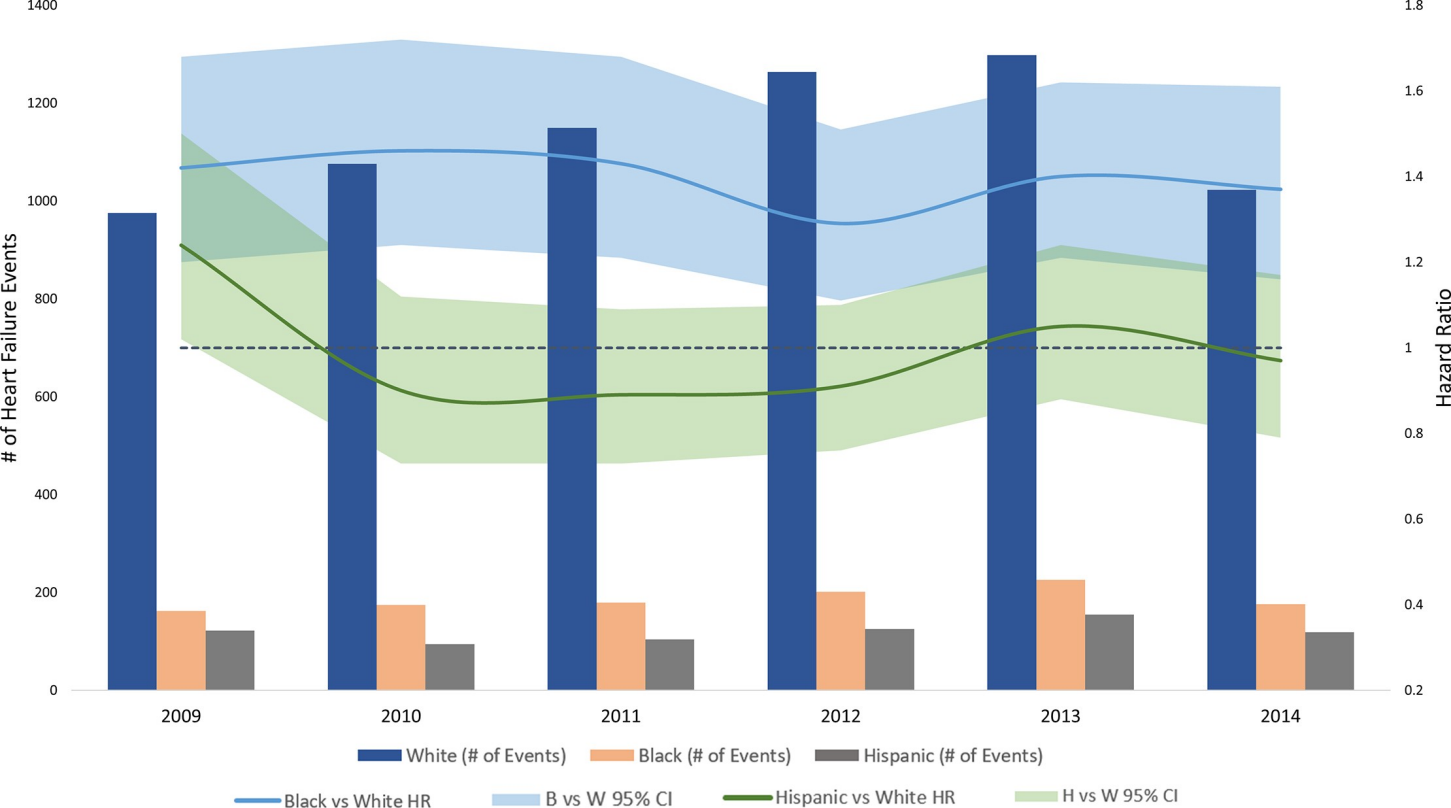
Population counts by race/ethnicity and adjusted HRs (95% CI) for associations of race/ethnicity with incidence of heart failure in patients with atrial fibrillation by year, Optum Clinformatics 2009–2015.

**Fig 6:**
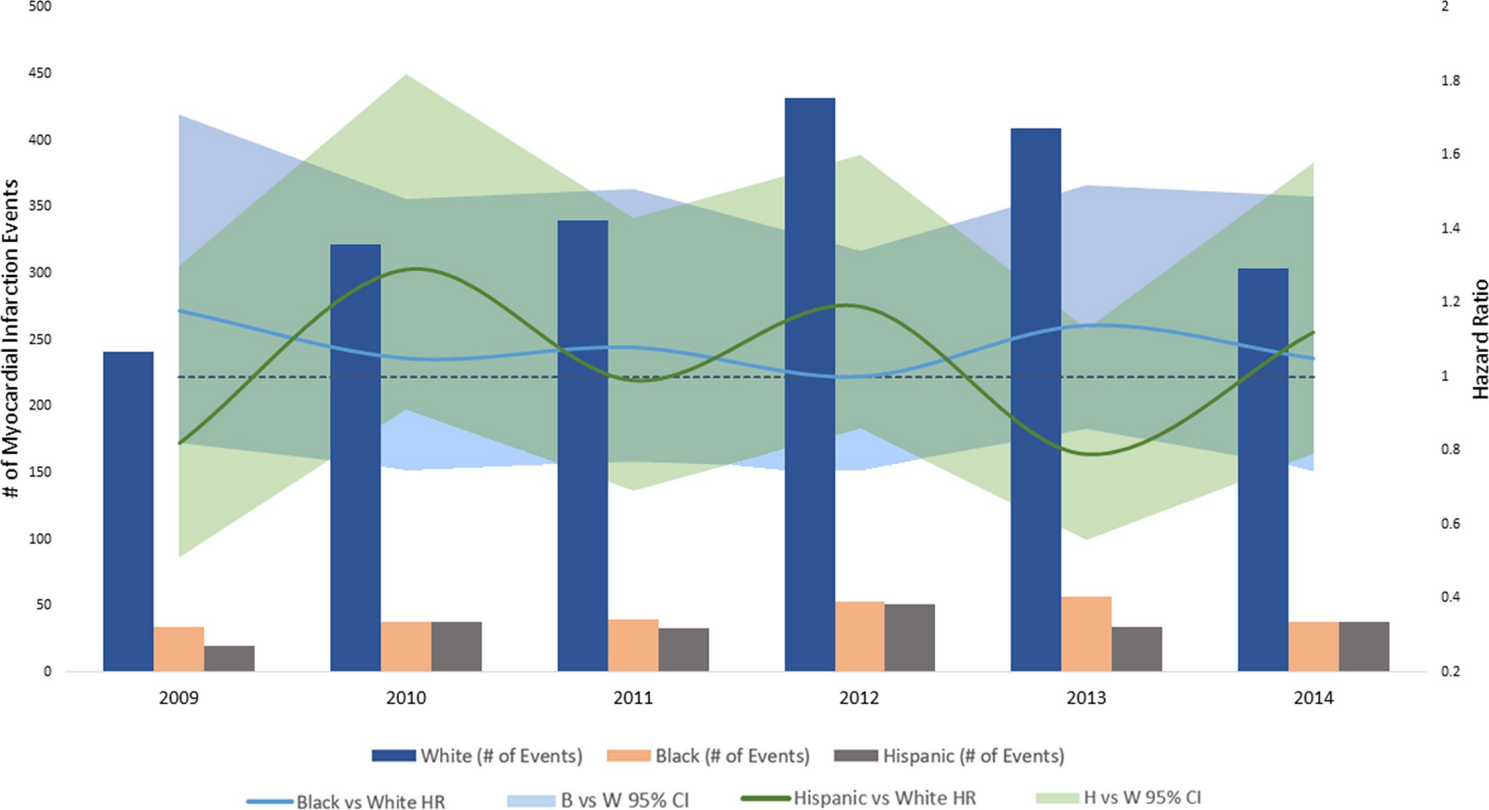
Population counts by race/ethnicity and adjusted HRs (95% CI) for associations of race/ethnicity with incidence of myocardial infarction in patients with atrial fibrillation by year, Optum Clinformatics 2009–2015.

In the analysis stratified by calendar year, women had consistently higher rates of ischemic stroke **(Fig 1)**, along with lower rates of heart failure **(Fig 2)** and myocardial infarction compared to men **(Fig 3)**. Compared to white patients, black patients had consistently higher rates of stroke **(Fig 4)** and heart failure **(Fig 5)**, while differences in rates of myocardial infarction over the years were small and consistent with the overall weak association between race and myocardial infarction **(Fig 6)**. Detailed numbers for these analyses are presented in **S5–S7 Tables**. There was less evidence of differences in the rates of outcomes for Hispanics compared to whites. Due to low counts within the database and for privacy protection, year-specific results for Asian Americans are not presented. Tests of interaction to determine whether sex or race/ethnicity are changing over time were not significant (**S5–S7 Tables**).

## Discussion

In this large administrative claims database in the United States, women with AF experienced a higher rate of ischemic stroke compared with men, while men had a higher rate of heart failure and myocardial infarction. Black patients with AF had a higher rate of ischemic stroke, heart failure, and myocardial infarction compared to whites, while no differences were observed between whites, Hispanics, and Asian Americans. These patterns held steady in year-stratified analysis from 2009 to 2014, as shown by no statistically significant interactions between year of diagnosis and race/sex, indicating lack of evidence that these differences have changed during this six-year period.

Recent reports have shown that women who have AF have a higher risk of ischemic stroke and myocardial infarction compared with men.[1] Our results were consistent with an increased risk for stroke, but we did not observe a higher risk of myocardial infarction in women. In the general United States population, men are more likely to develop coronary heart disease compared with women, [7] and this is also possibly true in our cohort of AF patients. Additionally, this may explain the higher risk for heart failure in men compared with women.

The data in this report also confirm that black patients with AF are more likely to develop cardiovascular disease events compared with whites. In the ARIC cohort, black patients with AF had a higher rate of stroke, heart failure, and coronary heart disease than whites.[2] Our findings further confirm this black-white difference in a large claims database and demonstrate that the rates of these outcomes do not vary between whites and other races/ethnicities.

The last decade has experienced important changes in the ability to manage AF, with the approval of non-vitamin K antagonist oral anticoagulants (NOACs) for stroke prevention and the more common use of catheter ablation for AF rhythm control.[8, 9] Unfortunately, management of AF—such as oral anticoagulation—is of lower quality in blacks compared to whites and in women compared to men in the US.[10–12] The lack of improvement in the differences in risk of stroke and other cardiovascular outcomes suggests that previously described treatment disparities are not being properly addressed.

Even though warfarin-use is on the downward trend in utilization among all racial/ethnic groups and sexes,[13] racial/ethnic and sex disparities are evident in NOAC use, with women with AF being less likely to use NOACs than men, and black and Hispanic patients with AF using NOACs at nearly half the rate of their white counterparts.[14] Total expenditure in the United States on NOACs increased from $95 million to $1.76 billion between 2010 and 2014 alone, with the out-of-pocket responsibility increasing from $119 million to $275 million during the same time period.[14]

These disparities also extend beyond medication. In a population of Medicare beneficiaries, women were less likely to visit electrophysiologists than men.[10] Between 2006 to 2011, women were less likely than men to undergo catheter ablation after hospital presentation with symptomatic AF, even though women experienced higher rates of AF-related rehospitalizations.[15] Similarly, blacks and Hispanics were far less likely to undergo catheter ablation compared to whites, yet experienced higher rates of rehospitalization.[15] These differences appear to remain persistent even after adjusting for insurance status and income.[15, 16] Such gaps in management often result in insufficient prophylaxis for endpoints such as stroke and heart failure.

Sex differences in cardiovascular outcomes have shown to be largely modified by age.[17, 18] In the early years, men are at higher risk of stroke compared to women, but in older age this association flips, with women not only being at higher risk of stroke, but also presenting with more severe symptoms.[19, 20] Our findings largely corroborate reports of elevated risk among older women for stroke. The observed age-sex interaction for women may partly be explained by the fact that women in the United States have longer life expectancies than men, extending the susceptibility period in which they could suffer strokes.[21] Additionally, the loss of estrogen following menopause may allow for the formation of atherosclerotic plaques, along with dysregulation of downstream kinase pathways that affect vascular reactivity.[22] This observation is consistent with the steep increase in overall cardiovascular risk in women following menopause. While female sex among AF patients seems to be a protective factor against heart failure and myocardial infarction, we did not find age to modify these associations. Previous studies have also confirmed an overall lower risk among women compared to men for heart failure and myocardial infarction hospitalizations, across AF status and regardless of age.[23–25]

Despite our study demonstrating an elevated risk of stroke, heart failure, and myocardial infarction in black AF patients compared to white AF patients, AF is not commonly diagnosed in blacks,[26] and has even been shown to be at lower risk of AF compared to other racial groups.[27] Risk factors associated with stroke are also more prevalent in black patients,[28] and there is evidence that AF in non-white populations may be more attributed to the higher prevalence of these risk factors, whereas white patients are at risk even in the absence of traditional risk factors and comorbidities.[29, 30] Furthermore, single nucleotide polymorphisms (SNPs) found to mediate a higher risk of AF in white Americans were not associated with increased risk of AF in black Americans.[31] European ancestry itself may even be risk factor for AF, including in black Americans.[32]

Our analysis should be interpreted in the context of its limitations. First, race may be misclassified in these data due to the imputation method, which has a sensitivity of less than 50% in classifying black race. However, we expect this misclassification to be non-differential with respect to the outcome and expect to bias our estimates of association towards the null.[33] Second, both AF and cardiovascular outcomes were based on diagnostic codes included in healthcare claims, which may lead to misclassification. However, whenever possible, we used validated algorithms with high positive predictive value to define AF and endpoints. If we assume that the misclassification was non-differential, the true effect would, in expectation, be larger than the observed effect. Third, results may not be generalizable to populations without health insurance. Finally, other factors associated with race and sex that were not available in claims records may confound the reported associations with cardiovascular outcomes.

Overall, the findings in this report indicate the presence of sex heterogeneity in the rate of adverse cardiovascular outcomes in patients with AF, confirm the adverse risk profile in blacks compared with whites who have AF, and highlight lack of progress in reducing those differences. Further research is needed to understand these findings in order to develop targeted preventive strategies to improve outcomes in these subgroups of AF patients and reduce overall cardiovascular health differences.

## Supporting information

S1 Table ICD-9-CM Codes for Covariates and Endpoint Diagnosis. (DOCX)

S2 Table Associations of sex and race/ethnicity with incidence of ischemic stroke in patients with atrial fibrillation, stratified by age, Optum Clinformatics 2009–2015. (DOCX)

S3 Table Associations of sex and race/ethnicity with incidence of heart failure in patients with atrial fibrillation, stratified by age, Optum Clinformatics 2009–2015. (DOCX)

S4 Table Associations of sex and race/ethnicity with incidence of myocardial infarction in patients with atrial fibrillation, stratified by age, Optum Clinformatics 2009–2015. (DOCX)

S5 Table Year-stratified incidences of ischemic stroke across race/ethnicity and sex in patients with atrial fibrillation, Optum Clinformatics 2009–2015. (DOCX)

S6 Table Year-stratified incidences of heart failure across race/ethnicity and sex in patients with atrial fibrillation, Optum Clinformatics 2009–2015. (DOCX)

S7 Table Year-stratified incidences of myocardial infarction across race/ethnicity and sex in patients with atrial fibrillation, Optum Clinformatics 2009–2015. (DOCX)

## References

[1] Ko D, Rahman F, Schnabel RB, Yin X, Benjamin EJ, Christophersen IE. Atrial fibrillation in women: epidemiology, pathophysiology, presentation, and prognosis. Nature reviews Cardiology. 2016;13(6):321–32. 10.1038/nrcardio.2016.45 27053455 PMC5579870

[2] Magnani JW, Norby FL, Agarwal SK, Soliman EZ, Chen LY, Loehr LR, et al Racial Differences in Atrial Fibrillation-Related Cardiovascular Disease and Mortality: The Atherosclerosis Risk in Communities (ARIC) Study. JAMA Cardiol. 2016;1(4):433–41. 10.1001/jamacardio.2016.1025 27438320 PMC5347977

[3] Wallace PJ, Shah ND, Dennen T, Bleicher PA, Crown WH. Optum Labs: building a novel node in the learning health care system. Health Aff (Millwood). 2014;33(7):1187–94. 25006145 10.1377/hlthaff.2014.0038

[4] Piccini JP, Hammill BG, Sinner MF, Jensen PN, Hernandez AF, Heckbert SR, et al Incidence and prevalence of atrial fibrillation and associated mortality among Medicare beneficiaries, 1993–2007. Circulation Cardiovascular quality and outcomes. 2012;5(1):85–93. 10.1161/CIRCOUTCOMES.111.962688 22235070 PMC3332107

[5] DeFrank JT, Bowling JM, Rimer BK, Gierisch JM, Skinner CS. Triangulating differential nonresponse by race in a telephone survey. Prev Chronic Dis. 2007;4(3):A60 17572964 PMC1955404

[6] Lip GY, Nieuwlaat R, Pisters R, Lane DA, Crijns HJ. Refining clinical risk stratification for predicting stroke and thromboembolism in atrial fibrillation using a novel risk factor-based approach: the euro heart survey on atrial fibrillation. Chest. 2010;137(2):263–72. 10.1378/chest.09-1584 19762550

[7] Benjamin EJ, Virani SS, Callaway CW, Chamberlain AM, Chang AR, Cheng S, et al Heart Disease and Stroke Statistics-2018 Update: A Report From the American Heart Association. Circulation. 2018;137(12):e67–e492. 10.1161/CIR.0000000000000558 29386200

[8] January CT, Wann LS, Alpert JS, Calkins H, Cigarroa JE, Cleveland JC Jr., et al 2014 AHA/ACC/HRS guideline for the management of patients with atrial fibrillation: executive summary: a report of the American College of Cardiology/American Heart Association Task Force on practice guidelines and the Heart Rhythm Society. Circulation. 2014;130(23):2071–104. 10.1161/CIR.0000000000000040 24682348

[9] January CT, Wann LS, Calkins H, Chen LY, Cigarroa JE, Cleveland JC Jr., et al 2019 AHA/ACC/HRS Focused Update of the 2014 AHA/ACC/HRS Guideline for the Management of Patients With Atrial Fibrillation. Circulation. 2019:CIR0000000000000665.

[10] Bhave PD, Lu X, Girotra S, Kamel H, Vaughan Sarrazin MS. Race- and sex-related differences in care for patients newly diagnosed with atrial fibrillation. Heart Rhythm. 2015;12(7):1406–12. 10.1016/j.hrthm.2015.03.031 25814418 PMC4787261

[11] Essien UR, Holmes DN, Jackson LR 2nd, Fonarow GC, Mahaffey KW, Reiffel JA, et al Association of Race/Ethnicity With Oral Anticoagulant Use in Patients With Atrial Fibrillation: Findings From the Outcomes Registry for Better Informed Treatment of Atrial Fibrillation II. JAMA Cardiol. 2018. 10.1001/jamacardio.2018.3945 PMC6583087 30484833

[12] Patel N, Deshmukh A, Thakkar B, Coffey JO, Agnihotri K, Patel A, et al Gender, Race, and Health Insurance Status in Patients Undergoing Catheter Ablation for Atrial Fibrillation. Am J Cardiol. 2016;117(7):1117–26. 10.1016/j.amjcard.2016.01.040 26899494

[13] Sur NB, Wang K, Di Tullio MR, Gutierrez CM, Dong C, Koch S, et al Disparities and Temporal Trends in the Use of Anticoagulation in Patients With Ischemic Stroke and Atrial Fibrillation. Stroke. 2019;50(6):1452–9. 10.1161/STROKEAHA.118.023959 31084325 PMC6538423

[14] Rodwin BA, Salami JA, Spatz ES, Valero-Elizondo J, Virani SS, Blankstein R, et al Variation in the Use of Warfarin and Direct Oral Anticoagulants in Atrial Fibrillation and Associated Cost Implications. Am J Med. 2019;132(1):61–70 e1. 10.1016/j.amjmed.2018.09.026 30290193

[15] Kummer BR, Bhave PD, Merkler AE, Gialdini G, Okin PM, Kamel H. Demographic Differences in Catheter Ablation After Hospital Presentation With Symptomatic Atrial Fibrillation. J Am Heart Assoc. 2015;4(9):e002097 10.1161/JAHA.115.002097 26396201 PMC4599497

[16] Tamariz L, Rodriguez A, Palacio A, Li H, Myerburg R. Racial disparities in the use of catheter ablation for atrial fibrillation and flutter. Clin Cardiol. 2014;37(12):733–7. 10.1002/clc.22330 25491888 PMC6647633

[17] Kuznetsova T. Sex Differences in Epidemiology of Cardiac and Vascular Disease. Adv Exp Med Biol. 2018;1065:61–70. 10.1007/978-3-319-77932-4_4 30051377

[18] Merz AA, Cheng S. Sex differences in cardiovascular ageing. Heart. 2016;102(11):825–31. 10.1136/heartjnl-2015-308769 26917537 PMC5993677

[19] Berglund A, Schenck-Gustafsson K, von Euler M. Sex differences in the presentation of stroke. Maturitas. 2017;99:47–50. 10.1016/j.maturitas.2017.02.007 28364868

[20] Shobha N, Sylaja PN, Kapral MK, Fang J, Hill MD, Investigators of the Registry of the Canadian Stroke N. Differences in stroke outcome based on sex. Neurology. 2010;74(9):767–71. 10.1212/WNL.0b013e3181d5275c 20194917 PMC2836873

[21] Laditka JN, Laditka SB. Stroke and active life expectancy in the United States, 1999–2009. Disabil Health J. 2014;7(4):472–7. 10.1016/j.dhjo.2014.06.005 25096630

[22] Haast RA, Gustafson DR, Kiliaan AJ. Sex differences in stroke. J Cereb Blood Flow Metab. 2012;32(12):2100–7. 10.1038/jcbfm.2012.141 23032484 PMC3519418

[23] Naser N, Kulic M, Dilic M, Dzubur A, Durak A, Pepic E, et al The Cumulative Incidence of Stroke, Myocardial infarction, Heart Failure and Sudden Cardiac Death in Patients with Atrial Fibrillation. Med Arch. 2017;71(5):316–9. 10.5455/medarh.2017.71.316-319 29284897 PMC5723164

[24] Movahed MR, Khan MF, Hashemzadeh M, Hashemzadeh M. Age adjusted nationwide trends in the incidence of all cause and ST elevation myocardial infarction associated cardiogenic shock based on gender and race in the United States. Cardiovasc Revasc Med. 2015;16(1):2–5. 10.1016/j.carrev.2014.07.007 25458070

[25] Rodriguez F, Wang Y, Johnson CE, Foody JM. National patterns of heart failure hospitalizations and mortality by sex and age. J Card Fail. 2013;19(8):542–9. 10.1016/j.cardfail.2013.05.016 23910583

[26] Staerk L, Sherer JA, Ko D, Benjamin EJ, Helm RH. Atrial Fibrillation: Epidemiology, Pathophysiology, and Clinical Outcomes. Circ Res. 2017;120(9):1501–17. 10.1161/CIRCRESAHA.117.309732 28450367 PMC5500874

[27] Chen ML, Parikh NS, Merkler AE, Kleindorfer DO, Bhave PD, Levitan EB, et al Risk of Atrial Fibrillation in Black Versus White Medicare Beneficiaries With Implanted Cardiac Devices. J Am Heart Assoc. 2019;8(4):e010661 10.1161/JAHA.118.010661 30741594 PMC6405685

[28] Amponsah MK, Benjamin EJ, Magnani JW. Atrial Fibrillation and Race—A Contemporary Review. Curr Cardiovasc Risk Rep. 2013;7(5). 10.1007/s12170-013-0327-8 PMC3815541 24198867

[29] Dewland TA, Olgin JE, Vittinghoff E, Marcus GM. Incident atrial fibrillation among Asians, Hispanics, blacks, and whites. Circulation. 2013;128(23):2470–7. 10.1161/CIRCULATIONAHA.113.002449 24103419

[30] Rodriguez CJ, Soliman EZ, Alonso A, Swett K, Okin PM, Goff DC Jr., et al Atrial fibrillation incidence and risk factors in relation to race-ethnicity and the population attributable fraction of atrial fibrillation risk factors: the Multi-Ethnic Study of Atherosclerosis. Ann Epidemiol. 2015;25(2):71–6, 6 e1. 10.1016/j.annepidem.2014.11.024 25523897 PMC4559265

[31] Roberts JD, Hu D, Heckbert SR, Alonso A, Dewland TA, Vittinghoff E, et al Genetic Investigation Into the Differential Risk of Atrial Fibrillation Among Black and White Individuals. JAMA Cardiol. 2016;1(4):442–50. 10.1001/jamacardio.2016.1185 27438321 PMC5395094

[32] Marcus GM, Alonso A, Peralta CA, Lettre G, Vittinghoff E, Lubitz SA, et al European ancestry as a risk factor for atrial fibrillation in African Americans. Circulation. 2010;122(20):2009–15. 10.1161/CIRCULATIONAHA.110.958306 21098467 PMC3058884

[33] Rothman K, Greenland S, Lash TL. Validity in epidemiologic studies Modern Epidemiology. 3rd ed. Philadelphia, PA: Lippincott Williams & Wilkins; 2008 p. 128–47.

