# Supplementary material for: Sex and racial differences in cardiovascular disease risk in patients with atrial fibrillation
Source: PLoS One. 2019 Sep 4;14(9):e0222147. doi: 10.1371/journal.pone.0222147 (PMC6726240; doi:10.1371/journal.pone.0222147)
Supplement: S1 Table — (DOCX) [file pone.0222147.s001.docx]

**S1 Table. ICD-9-CM Codes for Covariates and Endpoint Diagnosis.**

| **Outcome** | **ICD-9-CM Codes** |
| --- | --- |
| Diabetes mellitus | 250 |
| Ischemic Stroke | 433, 434, 435, 436, 437, 438 |
| Heart failure | 398.91, 402.01, 402.11, 402.91, 404.01, 404.03, 404.11, 404.13, 404.91, 404.93, 425.4, 425.9, 428 |
| Hypertension | 401, 402, 403, 404, 405 |
| Myocardial infarction | 410, 412 |
| Peripheral Artery Disease | 440.0, 440.2, 440.9, 443.9 |
