# Supplementary material for: Sex and racial differences in cardiovascular disease risk in patients with atrial fibrillation
Source: PLoS One. 2019 Sep 4;14(9):e0222147. doi: 10.1371/journal.pone.0222147 (PMC6726240; doi:10.1371/journal.pone.0222147)
Supplement: S2 Table — (DOCX) [file pone.0222147.s002.docx]

**S2 Table. Associations of sex and race/ethnicity with incidence of ischemic stroke in patients with atrial fibrillation, stratified by age, Optum Clinformatics® 2009-2015**

|  | **Men** | **Women** | **Whites** | **Blacks** | **Hispanics** | **Asian Americans** |
| --- | --- | --- | --- | --- | --- | --- |
| **N.** | 208,256 | 172,380 | 313,042 | 32,095 | 27,453 | 8,046 |
| **Age <=70** |  |  |  |  |  |  |
| **Person-Years of Follow-up** | 176,061 | 94,633 | 223,047 | 24,549 | 17,865 | 5,233 |
| **N. events** | 888 | 507 | 1,034 | 224 | 109 | 28 |
| **Crude IR^*^** | 5.0 | 5.4 | 4.6 | 9.1 | 6.1 | 5.4 |
| **HR (95%CI)^**^** | 1 (ref) | 0.96 (0.86, 1.07) | 1 (ref) | 1.90 (1.64, 2.21) | 1.26 (1.03, 1.54) | 1.20 (0.83, 1.75) |
|  |  |  |  |  |  |  |
| **70 < Age <= 80** |  |  |  |  |  |  |
| **Person-Years of Follow-up** | 126,923 | 106,563 | 192,478 | 17,570 | 18,425 | 5,013 |
| **N. events** | 1088 | 1103 | 1,713 | 244 | 178 | 56 |
| **Crude IR^*^** | 8.6 | 10.4 | 8.9 | 13.9 | 9.7 | 11.2 |
| **HR (95%CI)^**^** | 1 (ref) | 1.18 (1.09, 1.28) | 1 (ref) | 1.47 (1.28, 1.69) | 1.02 (0.87, 1.19) | 1.28 (0.98, 1.67) |
|  |  |  |  |  |  |  |
| **Age > 80** |  |  |  |  |  |  |
| **Person-Years of Follow-up** | 96,937 | 127,507 | 187,464 | 13,520 | 18,142 | 5,319 |
| **N. events** | 1275 | 2374 | 2924 | 300 | 338 | 87 |
| **Crude IR^*^** | 13.2 | 18.6 | 15.6 | 22.2 | 18.6 | 16.4 |
| **HR (95%CI)^**^** | 1 (ref) | 1.39 (1.30, 1.49) | 1 (ref) | 1.38 (1.23, 1.56) | 1.22 (1.09, 1.37) | 1.07 (0.86, 1.32) |
|  | Age-Sex Interaction | P <.0001 | Age-Race Interaction | P = 0.001 |  |  |

IR, incidence rate; HR, hazard ratio; CI, confidence interval.

^*^Per 1,000 person-years

^**^Cox model adjusted for age, sex, race/ethnicity, education and CHA_2_DS_2_-VASc scores
