# Supplementary material for: Sex and racial differences in cardiovascular disease risk in patients with atrial fibrillation
Source: PLoS One. 2019 Sep 4;14(9):e0222147. doi: 10.1371/journal.pone.0222147 (PMC6726240; doi:10.1371/journal.pone.0222147)
Supplement: S3 Table — (DOCX) [file pone.0222147.s003.docx]

**S3 Table. Associations of sex and race/ethnicity with incidence of heart failure in patients with atrial fibrillation, stratified by age, Optum Clinformatics® 2009-2015**

|  | **Men** | **Women** | **Whites** | **Blacks** | **Hispanics** | **Asian Americans** |
| --- | --- | --- | --- | --- | --- | --- |
| **N.** | 208,256 | 172,380 | 313,042 | 32,095 | 27,453 | 8,046 |
| **Age <=70** |  |  |  |  |  |  |
| **Person-Years of Follow-up** | 174,032 | 93,565 | 220,822 | 23,980 | 17,609 | 5,186 |
| **N. events** | 2,619 | 1420 | 2,920 | 749 | 307 | 63 |
| **Crude IR^*^** | 15.0 | 15.2 | 13.2 | 31.2 | 17.4 | 12.1 |
| **HR (95%CI)^**^** | 1 (ref) | 0.90 (0.84, 0.96) | 1 (ref) | 2.06 (1.90, 2.24) | 1.16 (1.03, 1.31) | 1.00 (0.78, 1.29) |
|  |  |  |  |  |  |  |
| **70 < Age <= 80** |  |  |  |  |  |  |
| **Person-Years of Follow-up** | 124,774 | 105,079 | 189,852 | 17,045 | 18,013 | 4,945 |
| **N. events** | 3074 | 2310 | 4,117 | 678 | 496 | 93 |
| **Crude IR^*^** | 24.6 | 22.0 | 21.7 | 39.8 | 27.5 | 18.8 |
| **HR (95%CI)^**^** | 1 (ref) | 0.87 (0.82, 0.92) | 1 (ref) | 1.65 (1.52, 1.79) | 1.19 (1.08, 1.31) | 0.88 (0.71, 1.08) |
|  |  |  |  |  |  |  |
| **Age > 80** |  |  |  |  |  |  |
| **Person-Years of Follow-up** | 94,615 | 125,276 | 183,680 | 13,145 | 17,820 | 5,245 |
| **N. events** | 3571 | 4264 | 6402 | 663 | 619 | 151 |
| **Crude IR^*^** | 37.7 | 34.0 | 34.9 | 50.4 | 34.7 | 28.8 |
| **HR (95%CI)^**^** | 1 (ref) | 0.89 (0.85, 0.93) | 1 (ref) | 1.33 (1.23, 1.45) | 0.96 (0.88, 1.04) | 0.83 (0.71, 0.98) |
|  | Age-Sex Interaction | P = 0.14 | Age-Race Interaction | P <.0001 |  |  |

IR, incidence rate; HR, hazard ratio; CI, confidence interval.

^*^Per 1,000 person-years

^**^Cox model adjusted for age, sex, race/ethnicity, education and CHA_2_DS_2_-VASc scores
