# Supplementary material for: Sex and racial differences in cardiovascular disease risk in patients with atrial fibrillation
Source: PLoS One. 2019 Sep 4;14(9):e0222147. doi: 10.1371/journal.pone.0222147 (PMC6726240; doi:10.1371/journal.pone.0222147)
Supplement: S4 Table — (DOCX) [file pone.0222147.s004.docx]

**S4 Table. Associations of sex and race/ethnicity with incidence of myocardial infarction in patients with atrial fibrillation, stratified by age, Optum Clinformatics® 2009-2015**

|  | **Men** | **Women** | **Whites** | **Blacks** | **Hispanics** | **Asian Americans** |
| --- | --- | --- | --- | --- | --- | --- |
| **N.** | 208,256 | 172,380 | 313,042 | 32,095 | 27,453 | 8,046 |
| **Age <=70** |  |  |  |  |  |  |
| **Person-Years of Follow-up** | 174,032 | 93,565 | 222,734 | 24,554 | 17,847 | 5,220 |
| **N. events** | 2,619 | 1420 | 1,189 | 182 | 124 | 29 |
| **Crude IR^*^** | 15.0 | 15.2 | 5.3 | 7.4 | 6.9 | 5.6 |
| **HR (95%CI)^**^** | 1 (ref) | 0.71 (0.64, 0.80) | 1 (ref) | 1.30 (1.11, 1.52) | 1.23 (1.02, 1.49) | 1.11 (0.77, 1.61) |
|  |  |  |  |  |  |  |
| **70 < Age <= 80** |  |  |  |  |  |  |
| **Person-Years of Follow-up** | 124,774 | 105,079 | 192,849 | 17,529 | 18,426 | 5,018 |
| **N. events** | 3074 | 2310 | 1,444 | 200 | 156 | 39 |
| **Crude IR^*^** | 24.6 | 22.0 | 7.5 | 11.4 | 8.5 | 7.8 |
| **HR (95%CI)^**^** | 1 (ref) | 0.89 (0.82, 0.98) | 1 (ref) | 1.40 (1.21, 1.63) | 1.10 (0.93, 1.31) | 1.05 (0.76, 1.44) |
|  |  |  |  |  |  |  |
| **Age > 80** |  |  |  |  |  |  |
| **Person-Years of Follow-up** | 94,615 | 125,276 | 188,320 | 13,597 | 18,312 | 5,369 |
| **N. events** | 3571 | 4264 | 1857 | 156 | 170 | 39 |
| **Crude IR^*^** | 37.7 | 34.0 | 9.9 | 11.5 | 9.3 | 7.3 |
| **HR (95%CI)^**^** | 1 (ref) | 0.78 (0.72, 0.85) | 1 (ref) | 1.08 (0.91, 1.27) | 0.91 (0.77, 1.06) | 0.74 (0.54, 1.01) |
|  | Age-Sex Interaction | P = 0.53 | Age-Race Interaction | P = 0.0002 |  |  |

IR, incidence rate; HR, hazard ratio; CI, confidence interval.

^*^Per 1,000 person-years

^**^Cox model adjusted for age, sex, race/ethnicity, education and CHA_2_DS_2_-VASc scores
