# Supplementary material for: Sex and racial differences in cardiovascular disease risk in patients with atrial fibrillation
Source: PLoS One. 2019 Sep 4;14(9):e0222147. doi: 10.1371/journal.pone.0222147 (PMC6726240; doi:10.1371/journal.pone.0222147)
Supplement: S5 Table — (DOCX) [file pone.0222147.s005.docx]

**S5 Table. Year-stratified incidences of ischemic stroke across race/ethnicity and sex in patients with atrial fibrillation, Optum Clinformatics® 2009-2015.**

|  | Men | Women | White | Black | Hispanic |
| --- | --- | --- | --- | --- | --- |
| **2009** |  |  |  |  |  |
| **N. Events** | 179 | 240 | 336 | 36 | 40 |
| **HR (95% CI)** | 1 (Ref) | 1.39 (1.14, 1.69) | 1 (Ref) | 1.01 (0.71, 1.44) | 1.29 (0.92, 1.81) |
| **2010** |  |  |  |  |  |
| **N. Events** | 217 | 270 | 372 | 58 | 47 |
| **HR (95% CI)** | 1 (Ref) | 1.30 (1.08, 1.55) | 1 (Ref) | 1.44 (1.09, 1.92) | 1.34 (0.97, 1.83) |
| **2011** |  |  |  |  |  |
| **N. Events** | 221 | 300 | 407 | 56 | 42 |
| **HR (95% CI)** | 1 (Ref) | 1.35 (1.13, 1.61) | 1 (Ref) | 1.39 (1.04, 1.85) | 1.14 (0.82, 1.57) |
| **2012** |  |  |  |  |  |
| **N. Events** | 254 | 317 | 451 | 63 | 45 |
| **HR (95% CI)** | 1 (Ref) | 1.33 (1.12, 1.57) | 1 (Ref) | 1.34 (1.03, 1.76) | 1.05 (0.77, 1.44) |
| **2013** |  |  |  |  |  |
| **N. Events** | 267 | 281 | 410 | 77 | 45 |
| **HR (95% CI)** | 1 (Ref) | 1.09 (0.92, 1.29) | 1 (Ref) | 1.72 (1.34, 2.21) | 1.06 (0.77, 1.45) |
| **2014** |  |  |  |  |  |
| **N. Events** | 181 | 231 | 326 | 40 | 34 |
| **HR (95% CI)** | 1 (Ref) | 1.30 (1.06, 1.58) | 1 (Ref) | 1.11 (0.80, 1.56) | 0.96 (0.67, 1.39) |
|  | Year-Sex Interaction | P = 0.17 | Year-Race Interaction | P = 0.31 |  |

HR, hazard ratio; CI, confidence interval.

^*^Cox model adjusted for age, sex, race/ethnicity, education and CHA_2_DS_2_-VASc.
