# Supplementary material for: Sex and racial differences in cardiovascular disease risk in patients with atrial fibrillation
Source: PLoS One. 2019 Sep 4;14(9):e0222147. doi: 10.1371/journal.pone.0222147 (PMC6726240; doi:10.1371/journal.pone.0222147)
Supplement: S6 Table — (DOCX) [file pone.0222147.s006.docx]

**S6 Table. Year-stratified incidences of heart failure across race/ethnicity and sex in patients with atrial fibrillation, Optum Clinformatics® 2009-2015.**

|  | Men | Women | White | Black | Hispanic |
| --- | --- | --- | --- | --- | --- |
| **2009** |  |  |  |  |  |
| **N. Events** | 686 | 592 | 975 | 162 | 122 |
| **HR (95% CI)** | 1 (Ref) | 0.90 (0.81, 1.01) | 1 (Ref) | 1.42 (1.20, 1.68) | 1.24 (1.02, 1.50) |
| **2010** |  |  |  |  |  |
| **N. Events** | 769 | 603 | 1076 | 174 | 94 |
| **HR (95% CI)** | 1 (Ref) | 0.84 (0.75, 0.93) | 1 (Ref) | 1.46 (1.24, 1.72) | 0.90 (0.73, 1.12) |
| **2011** |  |  |  |  |  |
| **N. Events** | 753 | 692 | 1149 | 179 | 104 |
| **HR (95% CI)** | 1 (Ref) | 0.93 (0.84, 1.04) | 1 (Ref) | 1.43 (1.21, 1.68) | 0.89 (0.73, 1.09) |
| **2012** |  |  |  |  |  |
| **N. Events** | 912 | 720 | 1264 | 201 | 125 |
| **HR (95% CI)** | 1 (Ref) | 0.87 (0.79, 0.96) | 1 (Ref) | 1.29 (1.11, 1.51) | 0.91 (0.76, 1.10) |
| **2013** |  |  |  |  |  |
| **N. Events** | 919 | 786 | 1298 | 226 | 155 |
| **HR (95% CI)** | 1 (Ref) | 0.90 (0.82, 0.99) | 1 (Ref) | 1.40 (1.21, 1.62) | 1.05 (0.88, 1.24) |
| **2014** |  |  |  |  |  |
| **N. Events** | 675 | 669 | 1023 | 176 | 119 |
| **HR (95% CI)** | 1 (Ref) | 1.08 (0.97, 1.21) | 1 (Ref) | 1.37 (1.16, 1.61) | 0.97 (0.79, 1.17) |
|  | Year-Sex Interaction | P = 0.07 | Year-Race Interaction | P = 0.89 |  |

HR, hazard ratio; CI, confidence interval.

^*^Cox model adjusted for age, sex, race/ethnicity, education and CHA_2_DS_2_-VASc.
