# Supplementary material for: Sex and racial differences in cardiovascular disease risk in patients with atrial fibrillation
Source: PLoS One. 2019 Sep 4;14(9):e0222147. doi: 10.1371/journal.pone.0222147 (PMC6726240; doi:10.1371/journal.pone.0222147)
Supplement: S7 Table — (DOCX) [file pone.0222147.s007.docx]

**S7 Table. Year-stratified incidences of myocardial infarction across race/ethnicity and sex in patients with atrial fibrillation, Optum Clinformatics® 2009-2015.**

|  | Men | Women | White | Black | Hispanic |
| --- | --- | --- | --- | --- | --- |
| **2009** |  |  |  |  |  |
| **N. Events** | 132 | 170 | 241 | 34 | 20 |
| **HR (95% CI)** | 1 (Ref) | 0.83 (0.65, 1.04) | 1 (Ref) | 1.18 (0.82, 1.71) | 0.82 (0.51, 1.30) |
| **2010** |  |  |  |  |  |
| **N. Events** | 234 | 173 | 322 | 38 | 38 |
| **HR (95% CI)** | 1 (Ref) | 0.81 (0.67, 0.99) | 1 (Ref) | 1.05 (0.75, 1.48) | 1.29 (0.91, 1.82) |
| **2011** |  |  |  |  |  |
| **N. Events** | 249 | 169 | 340 | 40 | 33 |
| **HR (95% CI)** | 1 (Ref) | 0.70 (0.57, 0.85) | 1 (Ref) | 1.08 (0.77, 1.51) | 0.99 (0.69, 1.43) |
| **2012** |  |  |  |  |  |
| **N. Events** | 316 | 234 | 432 | 53 | 51 |
| **HR (95% CI)** | 1 (Ref) | 0.83 (0.70, 0.98) | 1 (Ref) | 1.00 (0.75, 1.34) | 1.19 (0.86, 1.60) |
| **2013** |  |  |  |  |  |
| **N. Events** | 276 | 233 | 409 | 57 | 34 |
| **HR (95% CI)** | 1 (Ref) | 0.93 (0.78, 1.11) | 1 (Ref) | 1.14 (0.86, 1.52) | 0.79 (0.56, 1.13) |
| **2014** |  |  |  |  |  |
| **N. Events** | 213 | 175 | 304 | 38 | 38 |
| **HR (95% CI)** | 1 (Ref) | 0.90 (0.74, 1.11) | 1 (Ref) | 1.05 (0.75, 1.49) | 1.12 (0.79, 1.58) |
|  | Year-Sex Interaction | P = 0.34 | Year-Race Interaction | P = 0.82 |  |

HR, hazard ratio; CI, confidence interval.

^*^Cox model adjusted for age, sex, race/ethnicity, education and CHA_2_DS_2_-VASc.
